# Fistula in Ano: Stricture of the Rectum; Operation

**Published:** 1875-01-01

**Authors:** F. K. Bailey

**Affiliations:** Knoxville, Tenn.


### MEDICAL EXAMINER.

A

*Semi-Monthly Journal of Medical Sciences.*

EDITED BY N. S. DAVIS, M.D., AND F. H. DAVIS, M.D.

No. I. CHICAGO, JAN. 1, 1875. VOL. XVI.

### Original Communications.

### FISTULA IN ANO: STRICTURE OF THE RECTUM; OPERATION.

By F. K. BAILEY, M.D., KNOXVILLE, TENN.

W. H. E——; a stoutly-built man, about thirty. Bilious-nervous temperament, the latter predominating.

Was a sergeant in a Tennessee cavalry regiment during the late war.

Applied to me Aug. 23d, 1869, for examination in order to obtain a pension. Found two pile tumors upon the right side of the anus, with a purulent discharge from the rectum.

Considerable perineal swelling on the same side, with some redness and tenderness. Decided that a blind internal fistula existed, giving rise to purulent discharge. I made no examination of the rectum by exploration, as he lived twenty-five miles distant, and there was no time.

Sept. 11th. Requested to visit Mr. E——, at his residence. Arrived at 8 A. M., on the 12th, and found that an abscess on the left side of the perineum had opened externally, and about midway between the anus and the base of the scrotum. Pus escaping freely. Introduced a finger within the anus and found, about two inches above the sphincter, a stricture, consisting apparently of a band, and feeling like a rigid os uteri.

Passed a probe into the external opening, but failed to find it communicating with the rectal cavity.

On the right side of the anus there was tumefaction of the perineal cellular substance, with redness and pain, indicating that an abscess was forming.

Owing to the extreme soreness of the parts it was deemed inadvisable to interfere, except to apply emollient lotions, and to inject the bowel and the fistulous tract with warm water.

Remaining till next morning, I at that time placed a ligature around the pile tumor, and gave general directions for the management of the case as were indicated.

Sept. 19th. Made another visit and found much less soreness of the parts, but otherwise unchanged. The hæmorrhoidal tumors separated on the fifth day. Able to be about the house, but the discharge continues annoying.

Jan. 15th, 1870. Did not see Mr. E. till to-day, when he called at my office. Found a fistulous opening on the right side of the anus, near the verge, and at the point where there was swelling at last report. It had opened soon after and added much to the trouble. The stricture still remains, and on introducing a probe find the internal openings of the fistulous tracts to be above the band, and cannot be felt with the finger. Ex[illegible]ssive soreness of the parts externally, and passing the finger within the rectum causes great pain.

Informed him that nothing but surgical interference would promise any relief to his condition, and he decided to come to the city as soon as his business would admit, for treatment.

Feb. 10th, Mr. E. came to town and took a room at one of the hotels. There has been of late, as he says, torpidity of the bowels, with a sallow skin, and pain under the left scapula.

Feb. 12th. Bowels freely opened by taking comp. rhubarb pills. A quantity of hard feculent matter has been passed, relieving him very much. He can sit down with more ease than for some weeks, and the general tenderness relieved. To use enemata of warm water daily, and to have good diet, with a mild tonic.

Feb. 18th. The soreness having subsided so as to admit of manipulation, and, with the concurrence of Dr. Boyd, introduced a bivalve speculum, which revealed an ulcerated condition of the mucous membrane, with a free secretion of pus, which fills the speculum.

Very painful at the point of stricture, with the appearance of a septum or band from a portion of the coat of the intestine, besides which there appears to be another passage through which fœcal matter can pass.

Feb. 23d. Decided to make a thorough exploration, and accordingly he was placed under the influence of chloroform.

Present, Drs. Boyd and Boynton. On using considerable pressure the whole mass appeared yielding, and, with the introduction of first one, then two and three fingers, Dr. Boyd was able to break up the stricture and make an open passage. A large quantity of bloody pus and mucous tissue came away, with fœcal matter. Recovered from chloroform within an hour and felt comparatively free from pain. To have an anodyne dose of morphine, with enemata as required to wash out the rectum.

Feb. 24th. Vomited freely at midnight, but rested quite well with that one interruption. There had been a smothering sensation till after he vomited, caused probably by the chloroform. No more soreness than before the operation. Pulse full and about 90. Some appetite. Less discharge of pus, but copious fœcal stools have passed.

Feb. 25th. Returned to his home to-day and feels much relieved.

There had been some hesitation felt as to the propriety of breaking up the stricture, as it was uncertain what was its actual character. To allow it to remain longer hazarded retention of fœces, and pus constantly accumulated above it.

March 9th. My patient returned to town and was found to have improved somewhat in general health. The stricture cannot be felt to any extent, but there is a nodulated feeling to the inside of the rectum. The fistulous openings still unchanged. There is an indurated condition of the perineal tissue, which was unwelcome, but it was decided to attempt an operation.

March 12th. Dr. Boyd present. Chloroform administered by Dr. Rhea, of Newport. I introduced a grooved director into the opening on the left side, about one and a half inches, where it seemed to encounter an angular turn in the track. Dr. Boyd then passed a strong bistoury along the director and made an incision to the angular point.

Then it was attempted to pass a probe into the fistula opening upon the ischium, with a view to cut it out. This was upon the right side. It being about two and a half inches to the inner opening of the canal, and there being danger of injuring arteries in making an incision, it was concluded to pass a ligature and tie it. Accordingly a piece of surgeon's silk was doubled and well twisted, passed through the eye of the probe. The probe then was passed into the rectum, via the fistula, bent and brought out at the anus. A tight knot was tied and the ends clipped off. It was exceedingly difficult to manipulate in this case, as the patient was not completely under the influence of the chloroform, and it was nearly impossible to accomplish anæsthesia, on account of the unfavorable position, and the extreme tenderness of the parts. A full dose of opium was given, and water dressings ordered to be applied.

March 13th. Had some sleep, but complains much of soreness from the ligature. Bowels moved freely during the night, and without pain. To continue opium and soothing applications.

March 14th. Complains bitterly of soreness from the ligature. Slept but little, and none till injections of tr. opii. combined with hyposulphite of soda and carbolic acid, were used.

There was a free discharge of bloody pus this morning. To continue the enemata, and to apply cloths wet in the same preparation, externally. To have good food, with lager beer.

March 15th, 8 A. M. Pain kept him awake during most of last night, especially on moving about or coughing. Some appetite. Pulse 72 and soft. Tongue slightly coated white. To continue same treatment.

March 18th. There has been constant pain from the ligature till to-day, and there are indications that it is cutting its way slowly out through the included texture. Bowels moved daily, and without causing pain. Till to-day I have tightened the ligature every morning, but now conclude to allow a rest for two or three days. His appetite is good and he can eat heartily of ordinary fare. To take anodyne and continue the enemata.

March 20th. Quite comfortable. Pulse full and not frequent. Tongue cleaning. Gave a twist upon the ligature this morning which caused much pain. There is some discharge of pus from the ligated fistula, but less than formerly from the other side. The incised wound made to shorten the fistulous canal on the left side looks healthy. Bowels open. To be kept still in bed and have all the food he may desire. The sphincter is cut through by the ligature, and about half an inch of the subjacent tissue.

March 29th. The ligature still remaining. It has been gradually tightened, but only slight tension was admissible on account of the extreme soreness. Introduced my right index finger within the rectum and found more indications of stricture. A probe passed into the fistula alongside of the ligature, appears to enter for two and a half or three inches very readily, without going inside the intestine, and it must pass externally to the stricture band.

But very little pus escapes from the opening on the left side. Appetite good, and there has been but little pain for a few days past, except when the ligature was tightened.

April 2d. Went home to-day.

April 4th. Returned feeling no worse for his trip. Gave five comp. rhu. pills at night, to evacuate the bowels preparatory to an examination to-morrow.

April 5th, 4 P. M. Placed under chloroform. Dr. J. M. Boyd and Drs. J. M. Kennedy and C. Deaderick were present. I passed a grooved director within the fistula, to the rectum, and brought it out at the anus. Holding it firmly in that position Dr. Boyd passed a bistoury along the groove and cut the remaining portion of septum which had not been severed by the ligature. The internal opening was fully twenty lines from the sphincter, and large enough to admit the finger.

Since the application of the ligature an external pile tumor had formed, near the verge, posteriorly, and a little to the left side. At the base of this nipple-like projection was an opening which admitted a probe within the sphincter. This tumor was removed. Dr. Boyd then introduced his fingers to the rectum, and succeeded in breaking up the stricture which had re-formed at the original point. There was but very little bleeding from the cut tissue, or the crushed tissue at the stricture.

Twenty drops tr. opii. was at once given, and a sol. carbolic acid, tr. opii. and olive oil, placed within the incised part.

The fistula on the other side was not explored on this occasion.

April 10th. Since the 5th he has felt very well most of the time.

The incised part is disposed to gap, and seems disposed to heal from the bottom. Sanious pus escapes with every stool, as well as constantly passing for the want of retentive power. The condition of the other side is unchanged. There is a deep and tortuous fistulous tract or sinus which extends upwards two or three inches, and the tissue is indurated along the whole distance. Stools pass easily, but somewhat flattened or ribbon-shaped. Is able to walk about and is in very good spirits. Carbolic acid dressings.

April 16th. The open part slowly healing. The small fistulous opening still discharges pus from the bowel. There is a general indurated feel to the perineum, on all sides, with an unhealthy appearance, such as will render the healing process tedious.

Goes home to-day, with instructions to keep the bowels open with pills rhu. comp.; to inject with the female nozzle of a rubber syringe, sol. carbolic acid and hyposulphite soda.

May 6th. Mr. E. came to town, and I find the incised part has healed somewhat, but the cellular tissue is still indurated in the vicinity of all the fistulous tracts. The sinus on the left side, he says, is open still, as air passes out at times. He can pass the syringe tube beyond the stricture, and the enemata are retained. By constant use of the carbolic mixture he is able to keep the parts free from unpleasant smell, as well as cleanly. Appetite and strength improving. Looks better than at any time within the past year.

Jan. 4th, 1871. I have seen Mr. E. at times since May last, but made no examination till to day. There has been an improvement in the general health, but the fistulous openings still remain unchanged. On the right side there is a fissure at the point of incision, about half an inch deep. The unhealed surface has become somewhat callous, and the stools cannot be kept back. He is obliged to wear thick compresses in order to keep his clothing from being soiled, but manages to keep about and attend to an active out-door business.

At one time during the treatment of this case, in March, 1870, there appeared upon the chest and upper abdomen, some suspicious spots, which excited at least the possibility of a syphilitic taint. They soon disappeared after placing him upon iod. potassium a week or more, and, being a mere suspicion at the time, no note was made of the incident in my daily jottings in the case book.

Mr. E., up to a few weeks ago, (Nov., 1874) was able to attend to the duties of station agent on one of our railroads. I have seen him at times, but made no examination for two years at least. The condition is unchanged, as he informed me a few months ago, and he often speaks of coming to have surgical treatment. It has been suggested to close up the fissure, as in case of ruptured perineum, and, unless the tissue has become indurated, and in such a morbid condition as to refuse to heal when raw surfaces are drawn in close apposition, there is no doubt of success. Of the cancerous nature of the tissue involved in the stricture, I had not entertained a thought, and no microscopical examination was made of the *debris* after breaking up the contracted tissue. I had feared an incurable condition on account of the induration, which was more extended than would be expected from mere fistula. The occurrence of fistula in ano, and in perineo, should naturally presuppose a morbid state of the tissues in that region, and there is a traditionary idea, to say the least, that care should be exercised before an operation is proposed for a radical cure.

I am informed that the mother of Mr. E. is at present suffering from cancer of one of the breasts. At least she is under treatment for what a traveling cancer doctor calls cancer of the breast.

Disease of the rectum has been, under my observation of nearly forty years, rather uncommon, especially stricture.

Prof. Gross says, "it is oftener described than observed."

Ashton also remarks: "Those who have had the greatest opportunities, and the most extended fields for observation, whose acumen in the observation of disease and its diagnosis, and whose integrity is most to be relied on, have not met with this affection as a common occurrence." It is unnecessary to quote further on this point.

It may be considered as remarkable that so few cases of disease of the rectum are met with in civil and military practice. Cavalrymen are especially *liable* to injury in riding, but cases of actual disease of the rectum from that cause appear to be rare.

In circular No. 3, Surg. Gen'l's office, page 251, we find reports of four operations for fistula in ano, and two for hæmorrhoids (removal by ligature).

I operated once for fistula in the hospital at Quincy, Ills., but found but few cases of disease of the rectum. Mr. E., whose case is reported above, informs me that he was troubled with piles and soreness in the rectum after a long raid, in which they were kept in the saddle most of the time for about a week.

He remained in the service till near the close of the war, and, as I did not become cognizant of the case till Aug., 1869, considerable time elapsed before serious trouble ensued. The abscess which gave rise to the fistula did not occur till a short time before coming under my notice. Concerning the first commencement of the stricture there is no definite history.

There is a doubt, to say the least, whether causes subsequent to his leaving the army might or might not have operated in bringing on the condition in which I found him.

Should any further facts come to notice, or a future operation be performed, I will report.

